# Bidirectional association between autoimmune disease and perinatal depression: a nationwide study with sibling comparison

**DOI:** 10.1038/s41380-023-02351-1

**Published:** 2024-01-09

**Authors:** Emma Bränn, Yufeng Chen, Huan Song, Krisztina D. László, Brian M. D’Onofrio, Elgeta Hysaj, Catarina Almqvist, Henrik Larsson, Paul Lichtenstein, Unnur A. Valdimarsdottir, Donghao Lu

**Affiliations:** 1 Institute of Environmental Medicine, Karolinska Institutet, Stockholm, Sweden; 2 Department of Medical Epidemiology and Biostatistics, Karolinska Institutet, Stockholm, Sweden; 3 West China Biomedical Big Data Center, West China Hospital, Sichuan University, Chengdu, China; 4 Department of Global Public Health, Karolinska Institutet, Stockholm, Sweden; 5 Department of Public Health and Caring Sciences, Uppsala University, Uppsala, Sweden; 6 Department of Psychological and Brain Sciences, Indiana University, Bloomington, IN USA; 7 School of Medical Sciences, Örebro University, Örebro, Sweden; 8 Center of Public Health Sciences, Faculty of Medicine, University of Iceland, Reykjavík, Iceland

**Keywords:** Depression, Diseases

## Abstract

Although major depression, characterized by a pro-inflammatory profile, genetically overlap with autoimmune disease (AD) and the perinatal period involve immune system adaptations and AD symptom alterations, the bidirectional link between perinatal depression (PND) and AD is largely unexplored. Hence, the objective of this study was to investigate the bidirectional association between PND and AD. Using nationwide Swedish population and health registers, we conducted a nested case-control study and a matched cohort study. From 1,347,901 pregnancies during 2001–2013, we included 55,299 incident PND, their unaffected full sisters, and 10 unaffected matched women per PND case. We identified 41 subtypes of AD diagnoses recorded in the registers and compared PND with unaffected population-matched women and full sisters, using multivariable regressions. Women with an AD had a 30% higher risk of subsequent PND (95% CI 1.2–1.5) and women exposed to PND had a 30% higher risk of a subsequent AD (95% CI 1.3–1.4). Comparable associations were found when comparing exposed women with their unaffected sisters (nested case-control OR: 1.3, 95% CI 1.2–1.5, matched cohort HR: 1.3, 95% CI 1.1–1.6), and when studying antepartum and postpartum depression. The bidirectional association was more pronounced among women without psychiatric comorbidities (nested case-control OR: 1.5, 95% CI 1.4–1.6, matched cohort HR: 1.4, 95% CI 1.4–1.5) and strongest for multiple sclerosis (nested case-control OR: 2.0, 95% CI 1.6–2.3, matched cohort HR: 1.8, 95% CI 1.0–3.1). These findings demonstrate a bidirectional association between AD and PND independent of psychiatric comorbidities, suggesting possibly shared biological mechanisms. If future translational science confirms the underlying mechanisms, healthcare providers need to be aware of the increased risk of PND among women with ADs and vice versa.

## Introduction

Perinatal depression (PND), including antepartum (APD) and postpartum depression (PPD), affects approximately 3–20% of women worldwide [1]. Not only does PND affect the woman herself, but the child [2], the partner [3], and society [4]. Consequences of PND include sustained psychological ill-health, relationship difficulties, and lower quality of life [5]. However, studies investigating somatic diseases, including autoimmune diseases (ADs), in relation to PND are scarce [5].

Major depression outside the perinatal period has been linked to both endocrine-metabolic disorders [6] and dysfunctions of the immune system [7]. Moreover, mounting evidence supports the association between immune dysregulation and PND [8–15]. While women are at increased risk of AD during the first year after pregnancy [16], pregnancy has been shown to affect AD symptoms [17], where sensitivity to hormonal, physiological and psychological alterations in the perinatal period may contribute [18–20]. During pregnancy, type 1 T helper cell-related diseases, such as multiple sclerosis (MS) and rheumatoid arthritis, are often mitigated [21–23], whereas type 2 T helper cell-related diseases, such as systemic lupus erythematosus (SLE), commonly worsen [24].

Recently, a review suggested a bidirectional relation between PND and AD [25]. Although women with thyroid dysfunction [26–30] are at higher risk of PND, conflicting results have been reported for MS [31, 32]. Moreover, no study has comprehensively examined multiple ADs in relation to subsequent PND. Meanwhile, studies on risk of AD following PND are rather scarce. A higher risk of AD has been reported among women with perinatal psychiatric disorders [33] and women with PPD [34]. Yet, risk of AD after APD is unknown. Additionally, no studies have addressed shared risk factors (e.g., genetic factors) of PND and ADs, as emerging evidence reveals a sizeable genetic overlap between depression and AD [35].

Leveraging unique nationwide Swedish register data, we aimed to investigate the bidirectional association between PND and AD, namely risk of PND following AD as well as risk of AD after PND.

## Methods and materials

### Study design

We used the Swedish National Medical Birth Register (MBR) [36] to identify women (*n* = 825,401) who gave birth (*n* = 1,347,901) during 2001 to 2013 in Sweden. The MBR was established in 1973 and includes information on virtually all births in the country. Using the unique personal identification number assigned to every Swedish resident, these women were linked to the National Patient Register (NPR) [37] and the National Prescribed Drug Register (PDR) [38]. The NPR includes information from inpatient care since 1964 and specialized outpatient care since 2001. The PDR was established in 2005 and includes all prescribed drugs dispensed at pharmacies in Sweden. We identified full sisters of PND cases from the Multi-Generation Register, and deaths and emigrations during follow-up from the National Cause of Death Register and Migration Register. After excluding pregnancies with missing information on identification number or gestational age, multiple gestation, erroneous records, and pregnancies after PND diagnosis (as we only considered the first PND), 1,289,638 pregnancies from 815,232 women constituted our study base (Supplementary Fig. 1).

#### Nested case-control

To examine the association between AD and subsequent risk of PND we conducted a nested case-control study which preserves the statistical power and validity of a full cohort study, and yields estimates that are equivalent to the hazard ratio (HR) [39]. We identified all women with a first-ever PND as a depression diagnosis or antidepressant prescription recorded from the estimated date of conception to one year postpartum, in agreement with previous research [40–43]. Date of delivery and estimated gestational length were obtained from MBR to calculate the expected conception day. Using incidence density sampling, to each woman with PND we randomly sampled 10 women who were PND-free at the gestational age when APD was recorded or at the postpartum day when PPD was recorded. All unaffected women (No PND) were matched on age at delivery and calendar year of conception (for APD) or delivery (for PPD). In total, 55,299 PND (30,434 APD and 24,865 PPD) and 552,990 women with no PND were included (Supplementary Fig. 1). We then identified any diagnosis of AD before the matching date since 1981 when the NPR covered >80% of the inpatient care in the country.

#### Matched cohort

To investigate the association between PND and subsequent risk of AD, we conducted a matched cohort study. We excluded PND women with an AD before the matching date (*n* = 4,571) and their matched women with no PND (*n* = 42,882), and women with no PND but with an AD before the matching date (*n* = 30,439). This resulted in 50,728 PND exposed and 479,669 unexposed women for the cohort analysis (Supplementary Fig. 1). We followed the women from the matching date until diagnosis of AD, emigration, death, or December 31, 2013, and for unexposed women until a diagnosis of PND if any (thereafter the unexposed woman will contribute the time to the exposed group), whichever came first.

#### Sibling comparison

To further address unmeasured confounders shared by siblings, such as genetic and shared early life environmental factors, we conducted sibling comparison [44, 45], namely comparing the odds/risk of AD between a PND woman and her sister(s). To establish the sibling comparison study base, we restricted PND women to those who had at least one full and parous sister (to assure the sister was at risk of PND). Among the sisters of each PND woman, we randomly sampled one PND-free pregnancy from each sister for sibling comparison. Hence, when studying the association between ADs and subsequent risk of PND, the sibling analysis included 12,469 PND (6,797 APD and 5,672 PPD) and 14,549 no PND sisters. To study the association between PND and subsequent risk of AD, we further excluded PND women with AD before the matching date and their sisters, and no PND sisters with AD before the matching. In this analysis, 11,388 PND exposed (6,287 APD and 5,101 PPD) and 12,551 unexposed sisters were included (Supplementary Fig. 1). The follow-up started from the date of PND diagnosis for the PND women and the same gestational day/postpartum day for the sister(s).

### Measures

#### Ascertainment of PND

We defined PND as a depression diagnosis (Supplementary Table 1) recorded in the NPR or MBR, or a filled prescription of antidepressants (N06A) registered in PDR or MBR, between the expected date of conception and one year postpartum. If PND was ascertained through MBR only, the date of diagnosis/prescription was not available and the median date of pregnancy for each individual was assigned as date of diagnosis/prescription. PND was further sub-divided into a) APD if the first PND was recorded during pregnancy, and b) PPD if the first PND was recorded within 12 months after the delivery.

#### Ascertainment of AD

We searched the NPR for 41 first-ever diagnoses of ADs, as described by Song et al. [46] (Supplementary Table 1). The 41 ADs are included in classes of diseases of the endocrine system, inflammatory arthritis, vasculitis, connective tissue disorders, diseases of the skin system, hematological diseases, diseases of the nervous system, diseases of the digestive system, and others. However, the primary outcome was any AD. If more than one AD was present, the date of the earliest one was used.

#### Covariates

From the longitudinal integrated database for health insurance and labor market studies (LISA), the Total Population Register, and the National Medical Birth Register (MBR) we obtained information on maternal country of birth, and her personal disposable income, educational level, family situation, region of residence, and parity on the date for matching. Further, information on body mass index (BMI) in early pregnancy and smoking three months prior to pregnancy was retrieved from the MBR.

As a potential association could be driven from other psychiatric disorders, we identified any psychiatric diagnosis before the matching date from the National Patient Register (NPR) and classified them into depression, and other psychiatric disorders (Supplementary Table 1). If both depression and other diagnoses were present, the woman was classified into the depression category. To address possible pregnancy complications and outcomes that could mediate a potential association, we retrieved information on length of gestation, mode of delivery, and very premature delivery (<32 weeks) from the MBR, gestational diabetes, preeclampsia from the NPR (Supplementary Table 1) and stillbirth or neonatal death from the Causes of Death register. Missing values of all covariates were categorized as unknown. Categories of all covariates are presented in Supplementary Table 2.

### Statistical analysis

#### AD and risk of subsequent PND

In the nested case-control study, we used logistic regression, conditioning on matching set, to examine the association between overall AD and subsequent risk of PND and estimated odds ratios (ORs) with 95% confidence intervals (CI). In a next step we repeated our analyses with APD and PPD separately. In the sibling comparison, the logistic regression was conditioned on sibling set to control for familial factors shared by full sisters (e.g., genetic and familial environmental factors).

#### PND and risk of subsequent AD

In the matched cohort study, we used stratified Cox regression, conditioned on matching set, to examine the association between PND and subsequent AD and estimate HRs with 95% CIs. We tested the assumption of proportional hazards over time by plotting the Schoenfeld residuals for each variable and found no evidence of violation of this assumption. Consistent with the nested case-control analyses, Cox regression models were later repeated for APD and PPD separately, and in the sibling comparison.

We developed 4 models in both the population and the sibling analyses. In Model 0 we controlled for maternal age and calendar year of follow-up by stratifying on matching set in the population analysis and by multivariable adjustment in the sibling analyses. In Model 1, we additionally adjusted for demographics (maternal country of birth, educational level, region of residence, family situation, parity, and income). In Model 2, we additionally adjusted for shared risk factors between PND and AD, including BMI, smoking three months prior to pregnancy, and psychiatric comorbidity [47–52]. In Model 3, we additionally adjusted for pregnancy complications and outcomes including gestational diabetes, preeclampsia, mode of delivery, and length of gestation, which are risk factors for PPD and could be related to immune system activation [53–56]. As many covariates in Model 3 occurred after APD, we considered Model 2 as the primary model and therefore used in subsequent analyses. Due to comparable, yet less precise, associations obtained in the sibling comparison, only population analysis was performed in subsequent analyses.

#### Additional analyses

To assess the influence of prior diagnoses of psychiatric disorders, we conducted stratified analysis by psychiatric comorbidities. As Sweden introduced routine screening for postpartum depressive symptoms using the Edinburgh postnatal depression scale (EPDS) [57] in 2010 [58], presumably implying fewer underreported PND afterwards, we stratified the analysis by year of delivery before and after 2010. As immigrants may have difficulties to access healthcare and thus be more likely to have undiagnosed PND [59], we performed stratified analysis by maternal country of birth. In addition, we stratified the analyses by parity where the nature of incident PND was assured among primiparous women. To test for risk modification, an interaction term between PND and stratification factor was added and tested for statistical significance as *P* for interaction.

As AD is heterogeneous in etiology we performed analysis of type-specific AD to shed light on disease mechanism. Given data protection considerations, we only reported results for AD types with ≥10 outcome events.

To provide insights into timing of PND onset, analyses were conducted with APD separated into recorded in first, second, or last trimester, and PPD into <3, 3–6, and >6 months postpartum. To understand the recency on the link between AD and PND, we examined the associations by different timespans that AD occurred before or after PND.

Additionally, we excluded women who experienced stillbirth or neonatal death, or a very premature delivery (<32 weeks) as they can be considered potentially traumatic events that accounts for PPD. To further address possible misclassification of PND, we excluded PND cases identified by filled antidepressants only.

Data were processed using SAS 9.4 and analyzed using R (version 4.0.5). The study was approved by the Ethical Review Board in Stockholm, Sweden (2018/1515–31, 2012/1814-31/4, and 2013/862-31/5).

## Results

The mean age at diagnosis with PND was 30.7 (standard deviation 5.58) years. Compared to matched women with no PND, women with PND were more likely to be born in Scandinavia, have lower educational level, and to be single. They had higher BMI, were more likely to smoke, be primiparous, and to have pregnancy complications and psychiatric comorbidities. Further, PND was associated with premature delivery, cesarean section, and child loss. Similar patterns in background factors were observed when comparing PND women to their unaffected sisters (Supplementary Table 2).

### AD and risk of subsequent PND

In the nested case-control study, 8.3% of women with PND and 5.5% of matched women with no PND had an AD. After adjusting for demographics and pregnancy characteristics, ADs were associated with 30% higher risk of subsequent PND (OR: 1.30, 95% CI 1.25–1.35; Table 1, Model 2). Comparable associations were found when comparing sisters (PND: OR: 1.29, 95% CI 1.15–1.45, Table 1, Model 2) and when studying PPD and APD. We observed slightly attenuated, yet significant, associations when additionally controlling for pregnancy complications and outcomes (Supplementary Table 3; Model 3).

**Table 1:** Autoimmune disease and risk of subsequent perinatal depression: a population-based nested case-control study and sibling comparison.

Population										
Matching set	PND		No PND^a^		Model 1			Model 2		
	*n*	AD, *n* (%)	*n*	AD, *n* (%)	aOR	95% CI		aOR	95% CI	
PND	55,299	4571 (8.3)	552,990	30,439 (5.5)	**1.49**	**(1.44,**	**1.54)**	**1.30**	**(1.25,**	**1.35)**
APD	30,434	2217 (7.3)	304,340	14,766 (4.9)	**1.47**	**(1.40,**	**1.54)**	**1.26**	**(1.19,**	**1.33)**
PPD	24,865	2354 (9.5)	248,650	15,673 (6.3)	**1.52**	**(1.46,**	**1.60)**	**1.35**	**(1.28,**	**1.42)**
**Sibling**										
**Matching set**	**PND**		**No PND sister**		**Model 1**			**Model 2**		
	*n*	AD, *n* (%)	*n*	AD, *n* (%)	aOR	95% CI		aOR	95% CI	
PND	12,469	1081 (8.7)	14,549	859 (5,9)	**1.37**	**(1.24,**	**1.52)**	**1.29**	**(1.15,**	**1.45)**
APD	6797	510 (7.5)	7871	423 (5,4)	**1.30**	**(1.12,**	**1.50)**	1.18	(1.00,	1.41)
PPD	5672	571 (10.1)	6678	436 (6,5)	**1.46**	**(1.26,**	**1.68)**	**1.40**	**(1.18,**	**1.65)**

*AD* autoimmune disease, *PND* perinatal depression, *APD* antepartum depression, *PPD* postpartum depression, *aOR* adjusted odds ratio, *CI* confidence interval.

^a^Women matched to PND women at the diagnosis date of PND.

Values in bold indicate statistically significant results.

Model 1: the estimates were adjusted for age at delivery, year of delivery, maternal country of birth, region of residence, educational level, income, family situation, and parity.

Model 2: the estimates were additionally adjusted for body-mass index, smoking, and psychiatric comorbidity.

### PND and risk of subsequent AD

During a median follow-up of 4.75 years (maximum 13), we observed 3028 (6.0%) ADs among women exposed to PND and 18,323 (3.8%) among those unexposed to PND. Compared to women unexposed to PND, exposed to PND had a 30% higher subsequent risk of AD (HR: 1.30, 95% CI 1.25–1.36; Table 2, Model 2). Comparable results were obtained in the sibling analysis (PND: HR: 1.33, 95% CI 1.12–1.58; Table 2, Model 2), and when studying APD and PPD. The associations were somewhat attenuated, yet remained significant, when additionally adjusting for pregnancy complications and outcomes (Supplementary Table 3; Model 3).

**Table 2:** Perinatal depression and subsequent risk of autoimmune disease: a population-based matched cohort study and sibling comparison.

Population										
Matching set	Exposed to PND		Unexposed to PND^a^		Model 1			Model 2		
	*n*	AD, *n* (%)	*n*	AD, *n* (%)	aHR	95% CI		aHR	95% CI	
PND	50,728	3028 (6.0)	479,669	18,323 (3.8)	**1.52**	**(1.46,**	**1.58)**	**1.30**	**(1.25,**	**1.36)**
APD	28,217	1976 (7.0)	268,594	12,010 (4.5)	**1.50**	**(1.43,**	**1.58)**	**1.27**	**(1.20,**	**1.35)**
PPD	22,511	1052 (4.7)	211,075	6313 (3.0)	**1.55**	**(1.45,**	**1.65)**	**1.35**	**(1.25,**	**1.46)**
**Sibling**										
**Matching set**	**Exposed to PND**		**Unexposed to PND sister**		**Model 1**			**Model 2**		
	*n*	AD, *n* (%)	*n*	AD, *n* (%)	aHR	95% CI		aHR	95% CI	
PND	11,388	657 (5.8)	12,551	593 (4.7)	**1.45**	**(1.25,**	**1.68)**	**1.33**	**(1.12,**	**1.57)**
APD	6287	424 (6.7)	6896	380 (5.5)	**1.49**	**(1.23,**	**1.80)**	**1.33**	**(1.07,**	**1.66)**
PPD	5101	233 (4.6)	5655	213 (3.8)	**1.46**	**(1.13,**	**1.89)**	**1.44**	**(1.07,**	**1.95)**

*AD* autoimmune disease, *PND* perinatal depression, *APD* antepartum depression, *PPD* postpartum depression, *aHR* adjusted hazard ratio, *CI* confidence interval.

Values in bold indicate statistically significant results.

^a^Women matched to PND women at the diagnosis date of PND.

Model 1: the estimates were adjusted for age at delivery, year of delivery, maternal country of birth, region of residence, educational level, income, family situation, and parity.

Model 2: the estimated were additionally adjusted for body-mass index, smoking, and psychiatric comorbidity.

Stratified analyses showed that the bidirectional association between AD and PND was most pronounced among women without comorbidity of psychiatric disorders, whereas no/weaker association was noted among women with prior depression or other psychiatric disorders (p-for-interaction <0.001; Table 3).

**Table 3:** Associations between (a) autoimmune disease and risk of subsequent perinatal depression a nested case-control study, and (b) perinatal depression and subsequent risk of autoimmune disease a matched cohort study, stratified by psychiatric comorbidity.

(a) Autoimmune disease and subsequent risk of perinatal depression								
	PND		No PND^a^					
	*n*	AD, *n* (%)	*n*	AD, *n* (%)	aOR	95% CI	*p* for interaction	
By psychiatric comorbidity								
No	27,743	2166 (7.8)	505,318	26,511 (5.2)	**1.49**	**(1.42**	**1.56)**	**<0.001**
Depression	9262	772 (8.3)	7222	618 (8.6)	0.94	(0.84	1.06)	
Other disorders	18,296	1633 (8.9)	4045	3310 (8.2)	**1.12**	**(1.05**	**1.20)**	
**(b) Perinatal depression and subsequent risk of autoimmune disease**								
	**Exposed to PND**		**Unexposed to PND**^**a**^					
	*n*	AD, *n* (%)	*n*	AD, *n* (%)	aHR	95% CI	*p* for interaction	
By psychiatric comorbidity								
No	25,577	1448 (5.7)	439,513	16,499 (3.8)	**1.42**	**(1.35,**	**1.51)**	**<0.001**
Depression	849	520 (6.1)	605	294 (4.9)	0.88	(0.75,	1.03)	
Other disorders	16,661	1060 (6.4)	34,106	1530 (4.5)	**1.21**	**(1.11,**	**1.32)**	

The estimates were adjusted for age and calendar year at matching, maternal country of birth, income, educational level, body-mass index, region of residence, smoking, family situation, and parity.

*AD* autoimmune disease, *PND* perinatal depression, *aOR* adjusted odds ratio, *aHR* adjusted hazard ratio, *CI* confidence interval.

Values in bold indicate statistically significant results.

^a^Women matched to PND women at the diagnosis date of PND.

There was no evidence of effect modification by calendar year of birth (p-for-interaction >0.05; Supplementary Table 4). A stronger association between AD and subsequent PND was noted for women born outside Scandinavia than for those born in Scandinavia (p-for-interaction <0.001; Supplementary Table 4); whereas there was no evidence of effect modification when investigating the link between PPD and subsequent risk of AD (*p* for interaction >0.05; Supplementary Table 4). The bidirectional association was significant regardless of primiparity, although a slightly stronger association between PND and subsequent AD was noted for multiparous women (*p* for interaction <0.05; Supplementary Table 4).

### Types of ADs

In total, 27 types of ADs had ≥10 outcome events and 15 (55.5%) of them were significantly positively associated with PND in either direction. A significant positive bidirectional association was observed for autoimmune thyroid disease, psoriasis, MS, ulcerative colitis, and coeliac disease. Women with MS (OR: 1.96, 95% CI 1.64–2.34), Addison’s disease (OR: 1.93, 95% CI 1.15–3.24), and myasthenia gravis (OR: 1.90, 95% CI 1.02–2.54) had greatest increased risk of subsequent PND (Table 4). In the matched cohort study, highest relative risk was found for MS (HR: 1.79, 95% CI 1.03–3.11), Encephalitis, myelitis, and encephalomyelitis (HR: 1.51, 95% CI 1.20–1.89), and insulin dependent diabetes mellitus (HR: 1.45, 95% CI 1.37–1.55) (Table 4).

**Table 4:** Associations between (a) type-specific autoimmune disease and risk of subsequent perinatal depression in a nested case-control study, and (b) between perinatal depression and risk of subsequent type-specific autoimmune disease in a matched cohort study.

	(a) Autoimmune disease and risk of subsequent PND						(b) PND and risk of subsequent autoimmune disease					
	PND		No PND^a^				Exposed to PND		Unexposed to PND^a^			
	*n*	%	*n*	%	aOR	95% CI	*n*	%	*n*	%	aHR	95% CI
Endocrine												
Addison’s disease	21	0.04	103	0.02	**1.93**	**1.15–3.24**	NA					
Autoimmune thyroid disease	1573	2.84	10,558	1.91	**1.32**	**1.24–1.41**	1658	3.27	9242	1.93	**1.23**	**1.00–1.72**
Diabetes mellitus. insulin dependent	428	0.77	2723	0.49	1.06	0.94–1.19	90	0.18	505	0.11	**1.45**	**1.37–1.55**
Inflammatory arthritis												
Giant cell arteritis/ Polymyalgia rheumatica	25	0.05	112	0.02	**1.67**	**1.01–2.77**	14	0.03	40	0.01	1.00	0.72–1.40
Polyarteritis nodosa and related condition	27	0.05	138	0.02	**1.60**	**1.00–2.56**	NA					
Ankylosing spondylitis	80	0.14	431	0.08	**1.46**	**1.11–1.92**	54	0.11	287	0.06	1.11	0.76–1.61
Reactive arthritis (Reiter’s syndrome)	111	0.20	765	0.14	**1.34**	**1.07–1.67**	51	0.10	393	0.08	0.87	0.49–1.55
Henoch-Schonlein purpura	27	0.05	180	0.03	1.18	0.94–1.49	23	0.05	127	0.03		
Rheumatoid arthritis	247	0.45	2013	0.36	1.09	0.94–1.27	126	0.25	938	0.20	0.99	0.75–1.30
Connective tissue												
Behcet’s syndrome	22	0.04	91	0.02	**1.72**	**1.01–2.93**	NA					
Systemic lupus erythematosus	72	0.13	490	0.09	1.25	0.94–1.66	35	0.07	226	0.05	1.30	0.91–1.83
Mixed connective tissue disease	27	0.05	203	0.04	1.21	0.78–1.88	NA					
Systematic sclerosis (scleroderma)	16	0.03	206	0.04	0.62	0.36–1.06	NA					
SKIN												
Psoriasis	552	1.00	3586	0.65	**1.22**	**1.11–1.36**	476	0.94	2937	0.61	**1.22**	**1.09–1.36**
Vitiligo	65	0.12	489	0.09	1.21	0.90–1.63	37	0.07	332	0.07	0.90	0.59–1.37
Alopecia areata	23	0.04	139	0.03	1.18	0.72–1.96	17	0.03	128	0.03	1.35	0.89–2.05
Hematological												
Idiopathic thrombocytopenic purpura	47	0.08	403	0.07	1.01	0.72–1.42	18	0.04	182	0.04	1.61	0.71–3.68
Pernicious anemia	11	0.02	48	0.01	2.06	0.97–4.36	NA					
Nervous system												
Multiple sclerosis	200	0.36	909	0.16	**1.96**	**1.64–2.34**	78	0.15	591	0.12	**1.79**	**1.03–3.11**
Myasthenia gravis	20	0.04	61	0.01	**1.90**	**1.02–3.54**	NA					
Encephalitis. myelitis and encephalomyelitis	27	0.05	180	0.03	1.50	0.96–2.35	23	0.05	127	0.03	**1.51**	**1.20–1.89**
Guillain-Barré syndrome	18	0.03	135	0.02	1.25	0.72–2.17	NA					
Digestive												
Ulcerative colitis	400	0.72	2811	0.51	**1.34**	**1.19–1.51**	177	0.35	1076	0.22	**1.29**	**1.03–1.62**
Crohn’s disease	307	0.56	2060	0.37	**1.24**	**1.08–1.42**	117	0.23	591	0.12	1.10	0.89–1.37
Coeliac disease	437	0.79	2903	0.52	**1.23**	**1.10–1.38**	119	0.23	692	0.14	**1.35**	**1.12–1.63**
Other												
IgA nephropaty	137	0.25	850	0.15	**1.27**	**1.03–1.56**	39	0.08	268	0.06	1.24	0.66–2.32
Sarcoidosis	64	0.12	385	0.07	1.16	0.86–1.56	40	0.08	293	0.06	0.88	0.60–1.31

For both analyses we present number of individual AD and the percentage of AD among individuals with and without PND, respectively. The estimates were adjusted for age and calendar year at matching. maternal country of birth. income. educational level. BMI. geographic area of residence. smoking. marital status. parity. and psychiatric comorbidity.

*NA* not applicable due to <10 individuals.

Values in bold indicate statistically significant results.

^a^Women matched to PND women at the diagnosis date of PND.

Regarding recency of AD to/from PND, robust bidirectional associations were found over time. AD diagnosed within one year before or after matching was most strongly associated with PND (Supplementary Table 5). Regarding timing of PND onset, positive associations were noted for all APD and PPD groups, except for risk of AD associated with PPD within 3 months postpartum (Supplementary Table 6).

In sensitivity analyses, comparable bidirectional associations were obtained after excluding PND women with adverse pregnancy outcomes. Excluding PND identified through filled antidepressants only attenuated the risk for subsequent PND (OR: 1.17, 95% CI 1.09–1.26), but the association between PND and subsequent AD remained unchanged (HR: 1.30, 95% CI 1.21–1.39) (Supplementary Table 7).

## Discussion

In this nationwide register-based study in Sweden, we found a robust bidirectional association between PND and AD. Women with AD had an increased risk of subsequent PND, while exposure to PND was associated with an increased risk of subsequent AD. Our findings were further confirmed in the sibling comparison, where shared genetic and early life familial environmental factors were controlled for. The bidirectional association was independent of psychiatric comorbidities and was most pronounced among women without psychiatric comorbidities.

To our knowledge, this is the first study to show an elevated risk of PND following overall AD. Elevated risk for depression outside the perinatal period following overall ADs (23 disease-types studied) has previously been suggested [60]. For the other direction (PND-AD), our study is the most comprehensive, entailing 41 types of ADs. Lin et al. [34] reported that PPD was associated with a 61% increased risk of subsequent AD overall (31 disease-types studied). Brown et al. [33] found a 54% higher risk of AD overall (29 disease-types) in women with perinatal mental disorders. Our findings are in line with both studies, although with a weaker association possibly due to adjustment of more confounders, and are supported by studies outside the perinatal period [61].

Non-perinatal depression or other psychiatric disorders have been associated with PND [51] and ADs [61]. It is therefore plausible that psychiatric comorbidities underlie the observed bidirectional association. However, we found the association most pronounced among women without a comorbid psychiatric disorder or depression, suggesting that our findings cannot be completely explained by psychiatric comorbidities. Importantly, we observed a moderate bidirectional association between PND and AD among women with a history of any psychiatric disorders (other than depression) indicating that PND contributes to additional risk of AD to that of other psychiatric disorders, and vice versa. Previous studies typically excluded PND women with a history of psychiatric disorder. Among women with a history of depression, PND did not add on to the major depression-AD link, possibly due to an already altered inflammatory state imposed by major depression [7]. In women without a history of depression, PND is possibly linked to AD in a similar way as major depression is linked, namely through immune dysregulation [62]. In addition, such link may be modulated by major alterations of immune response occurring during pregnancy and postpartum [63]. Evidence suggests major depression having genetic overlap with various ADs [35], which may explain the observed bidirectional association [60]. However, the results were materially unchanged in sibling comparison suggesting that genetic overlap between PND and AD is less likely to explain our findings.

Literature supports varying pathophysiological pathways in PND [64, 65]. In this study, we analyzed APD and PPD separately, providing insight to APD that has not been studied thoroughly before. Studies suggest PND with onset within the first months postpartum to be associated with the withdrawal of hormones [66] and to be genetically different [67] from PND at other timepoints. However, we find the weakest association in the nested case-control study and null association in the matched-cohort study for PPD diagnosed in early postpartum period. Of note, we defined the time window based on date of diagnosis, which might be significantly later than the time of symptom onset. That said, some “early-onset” PPD could be diagnosed 4–6 months postpartum or even later. Moreover, caution should be exercised when interpreting these results given the overlapping CIs. Future studies with precise information on symptom onset are needed to better understand the interplay between hormone, immune profile, and psychiatric sensitivity. Further, we find point estimates suggesting stronger associations for PPD than APD. Some common ADs (such as RA) go into remission during pregnancy (possibly due to the downregulation of type 1 T helper cell activity [68]) and flare in the postpartum period, which could increase the risk of subsequent PPD rather than APD [69]. Regarding the other direction, previous studies suggest women with APD to have a profile of downregulated mostly anti-inflammatory immune markers, while women with PPD display upregulated pro-inflammatory markers [10, 12]. This upregulation of pro-inflammatory markers in the postpartum period in women with PPD could potentially serve as a trigger for subsequent ADs. However, in this study CIs and analyses of different timing of diagnoses, suggested no statistical differences in the association of ADs and PND across the perinatal period. Hence, our results may not fully support the pathophysiological differences between APD and PPD.

We found that about 56% of AD types were positively associated with PND in either direction. Previous studies are inconclusive of a higher risk of PND in women with MS, some reporting a bidirectional link [31] while others do not find women with MS to have elevated risk for PND [32]. However, increased prevalence rate of depression in MS patients outside the perinatal period has been reported [70]. A bidirectional association has been suggested between PPD and autoimmune thyroid disease before [26–30, 71]. Our results of psoriasis, ulcerative colitis, and coeliac disease, are in line with previous findings [25, 34, 72–74] but further add to the knowledge base by showing that PND is bidirectionally associated. Future research investigating these diseases, including possible disease mechanisms that likely differ across AD subtypes, is warranted.

During pregnancy, alterations in hormonal levels occur [75], including higher levels of estrogens with both pro- and anti-inflammatory capacity [76] and lower levels of progesterone that dampens inflammation [77]. The inflammatory profile of women with AD might increase sensitivity to these hormonal changes [78], contributing to increased risk of PND. Further, some types of ADs, e.g., SLE, are associated with increased risk of pregnancy complications [79], requiring clinical management before and throughout pregnancy. This could induce a stress response which could add to the risk of PND [80]. Besides new routines and sleep disruptions associated with transition to parenthood, sensitivity to the drop of gonadotropin and stress-related hormones after delivery [75] might contribute to both PPD and to the exacerbation of ADs. Furthermore, women with PND are at risk of long-term psychiatric complications [81], linked to inflammation [82], which could contribute to higher risk of AD years later. Notably, in the association between PND and subsequent AD, several ADs have a prodromal period of 5–10 years comprising of increased inflammation and unspecific symptoms, which can include depressive symptoms [83, 84]. Hence, the association between PND and subsequent AD may be explained by the prodromal pathobiology of some ADs. However, we found a somewhat greater HR of AD at follow-up >10 years compared to 5–10 years, suggesting that our findings cannot be completely explained by prodromal time. Moreover, a prodromal period will not explain the association found in the nested case-control study.

Strengths of this study include the population-based design with large sample size, prospectively collected data, and complete and long follow-up. Furthermore, sibling comparison allows controlment for genetic and familial factors. This study has some limitations. Women with AD are more likely to be monitored throughout pregnancy. However, concern of surveillance bias is largely alleviated by the robust associations yielded after introducing routine EPDS screening in 2010, and AD diagnosed >10 years before or after matching being associated with PND. We might have missed PND diagnosed in primary care during 2001–2005, and afterwards for women without antidepressant prescription. However, such misclassification would have led to attenuated associations. Some PND might not be incident, particularly for those who have given birth before 2001 where we don’t have data on outpatient records. However, the bi-directional association was noted in primiparous women. Antidepressants can be used for reasons other than depression. However, comparable findings were obtained by excluding PND identified through antidepressants only. Further, we were not able to define psychiatric comorbidities through outpatient care before 2001 and through prescribed antidepressants before 2005. In this context, the covariate - psychiatric comorbidity - rather represents currently comorbid conditions. In addition, misclassification of ADs is possible. However, studies have shown fairly good quality of several ADs e.g. myasthenia gravis [85] and MS [86], in the NPR. Last, women with severe AD may decide not to get pregnant and, by definition, are not part of our study population.

Our findings suggest a bidirectional association between PND and AD, independent of psychiatric comorbidities. These findings have implications for research on biological mechanisms, and for healthcare professionals who need to be alert to the risk of PND in women with AD, and vice versa.

### Supplementary information

SUPPLEMENTAL MATERIAL

## Data Availability

Swedish register data, due to privacy protection governed by the General Data Protection Regulation (GDPR), can only be accessed after granted ethical approval by appropriate authorities. Information regarding data access to Swedish register data can be found at the Swedish National Board of Health and Welfare (https://bestalladata.socialstyrelsen.se/, email: registerservice@socialstyrelsen.se) and/or Statistics Sweden (https://www.scb.se/vara-tjanster/bestall-data-och-statistik/, email: scb@scb.se). Codes for data analysis can be shared upon request to the corresponding author.
