# Correction: Bidirectional association between autoimmune disease and perinatal depression: a nationwide study with sibling comparison

**DOI:** 10.1038/s41380-024-02438-3

**Published:** 2024-02-06

**Authors:** Emma Bränn, Yufeng Chen, Huan Song, Krisztina D. László, Brian M. D’Onofrio, Elgeta Hysaj, Catarina Almqvist, Henrik Larsson, Paul Lichtenstein, Unnur A. Valdimarsdottir, Donghao Lu

**Affiliations:** 1 Institute of Environmental Medicine, Karolinska Institutet, Stockholm, Sweden; 2 Department of Medical Epidemiology and Biostatistics, Karolinska Institutet, Stockholm, Sweden; 3 West China Biomedical Big Data Center, West China Hospital, Sichuan University, Chengdu, China; 4 Department of Global Public Health, Karolinska Institutet, Stockholm, Sweden; 5 Department of Public Health and Caring Sciences, Uppsala University, Uppsala, Sweden; 6 Department of Psychological and Brain Sciences, Indiana University, Bloomington, IN USA; 7 School of Medical Sciences, Örebro University, Örebro, Sweden; 8 Center of Public Health Sciences, Faculty of Medicine, University of Iceland, Reykjavík, Iceland

**Keywords:** Depression, Diseases

Correction to: *Molecular Psychiatry* 10.1038/s41380-023-02351-1, published online 09 January 2024

In Table 4 of this article, in the column headed No PND^a^, n, on the row headed Diabetes mellitus. insulin dependent, the value ‘2.723’ should have read ‘2723’.

In Table 4 of this article, in the column headed No PND^a^, n, on the row headed Crohn’s disease, the value ‘2,06’ should have read ‘2060’.

The original article has been corrected.

