# Supplementary material for: Bidirectional association between autoimmune disease and perinatal depression: a nationwide study with sibling comparison
Source: Mol Psychiatry. 2024 Jan 9;29(3):602–10. doi: 10.1038/s41380-023-02351-1 (PMC11153129; doi:10.1038/s41380-023-02351-1)
Supplement: Supplementary file 1 — SUPPLEMENTAL MATERIAL [file 41380_2023_2351_MOESM1_ESM.docx]

## Supplementary information:

Bidirectional association between autoimmune disease and perinatal depression: a nationwide study with sibling comparison

Emma Bränn^1^, Yufeng Chen^1,2^, Huan Song^2,3^, Krisztina D. László^4,5^, Brian M. D’Onofrio^2,6^, Elgeta Hysaj^1^, Catarina Almqvist^2^, Henrik Larsson^2,7^, Paul Lichtenstein^2^, Unnur A. Valdimarsdottir*^1,8^, Donghao Lu*^1^

^* Equal contribution^

1. Institute of Environmental Medicine, Karolinska Institutet, Stockholm, Sweden
2. Department of Medical Epidemiology and Biostatistics, Karolinska Institutet, Stockholm, Sweden
3. West China Biomedical Big Data Center, West China Hospital, Sichuan University, Chengdu, China
4. Department of Global Public Health, Karolinska Institutet, Stockholm, Sweden
5. Department of Public Health and Caring Sciences, Uppsala University, Uppsala, Sweden
6. Department of Psychological and Brain Sciences, Indiana University, USA
7. School of Medical Sciences, Örebro University, Örebro, Sweden
8. Center of Public Health Sciences, Faculty of Medicine, University of Iceland, Reykjavík, Iceland

Summary:

This information contain the study flowchart, ICD-codes used, background characteristics, all four adjusted models, and stratified analyses by calendar year, country of birth, and parity. Further, time of autoimmune disease to/from PND, and timing of PND diagnoses. Lastly, sensitivity analysis excluding women with grief/stressors, and antidepressants defined PND.

#

Supplementary Figure 1. Flowchart of population and sibling cohort. The date of perinatal depression (PND) diagnosis/prescription of antidepressant medication is assigned as the matching date. For unaffected sisters, same gestational/postpartum day as PND diagnosis/prescription of antidepressant medication of the PND sister, is assigned as the matching date.

| Supplementary Table 1. Swedish International Classification of Diseases codes used to identify psychiatric, pregnancy and autoimmune diseases. | | | |
| --- | --- | --- | --- |
|  | **ICD-8** | **ICD-9** | **ICD-10** |
| **Psychiatric diseases** |  |  |  |
| PND | **-** | **-** | F32, F33, F53.0 |
| Depression | 3004 | 300E, 311 | F32-F33 |
| Other psychiatric | 290-315 | 290-319 | F (excl. F32-F33) |
| **Pregnancy diseases** |  |  |  |
| Preeclampsia | 63703-99 | 642E-G | O14-15 |
| Gestational diabetes | - | 648W | O244 |
| **Autoimmune diseases** | **ICD-8** | **ICD-9** | **ICD-10** |
| ***Disease of endocrine system*** |  |  |  |
| Diabetes mellitus, insulin dependent | 250 | 250 | E10 |
| Autoimmune thyroid disease | 242.00, 242.09, 244, 245.03 | 242A,  242X, 244W, 244X, 245C, 245W | E03.5, E03.9, E05.0, E05.5, E05.9, E06.3, E06.5 |
| Addison’s disease | 255.1 | 255E | E27.1, E27.2 |
| Autoimmune polyglandular syndrome | 258.10 | 258B | E31.0 |
| ***Inflammatory arthritis*** |  |  |  |
| Reactive arthritis (Reiter’s syndrome) | 099.3, 711.1 | 099D, 711A | M02.3, M02.8, M02.9 |
| Rheumatoid arthritis | 712.00, 712.10, 712,20, 712,39, 712.38 | 714A-D, 719D | M05, M06, M08.0, M08.1, M08.2, M08.3, M08.4 |
| Ankylosing spondylitis | 712.4 | 720A | M45 |
| **Vasculitis** |  |  |  |
| Polyarteritis nodosa and related condition (Incl. Kawasaki, Churg-Strauss syndrome, etc.) | 446 | 446A, 446B | M30 |
| Thrombotic microangiopathy | - | 446G | M31.1 |
| Granulomatosis with polyangiitis (Wegeners’s granulomatosis) | 446.20 | 446E | M31.3 |
| Microscopic polyangiitis | - | - | M31.7 |
| Henoch-Schönlein purpura | 287.00 | 287A | D69.0 |
| Giant cell arteritis/ Polymyalgia rheumatica | 446.30,446.31,446.39,717.9 | 446F, 725 | M35.3, M31.5, M31.6 |
| ***Connective tissue disorders*** |  |  |  |
| Systemic lupus erythematosus | 734.1 | 710A | M32 |
| Polymyositis/dermatomyositis | 716.10, 716.00 | 710E, 710D | M33.0, M33.1, M33.2, M33.9 |
| Systematic sclerosis (scleroderma) | 710.0, 734.0 | 710B | M34 |
| Sjögren’s syndrome | 734.90 | 710C | M35.0 |
| Mixed connective tissue disease | 734.98, 734.91, 734.99 | 710X, 710W | M35.1 |
| Behcet’s syndrome | 136.0 | 136B | M35.2 |
| ***Disease of skin system*** |  |  |  |
| Pemphigus vulgaris | 694.00 | 694E | L10.0 |
| Bullous pemphigoid | 694.05 | 694F | L12 |
| Dermatitis herpetiformis | 693.99 | 694A | L13.0 |
| Psoriasis | 696 | 694A, 696B | L40 |
| Alopecia areata | 704.00 | 704A | L64 |
| Vitiligo | 709.05 | 709A | L80 |
| ***Hematological diseases*** |  |  |  |
| Pernicious anemia | 281.0 | 281A | D51.0 |
| Autoimmune hemolytic anemia | 283.90, 283.92 | 283A | D59.0, D59.1 |
| Idiopathic thrombocytopenic purpura | 287.3 | 287D | D69.3 |
| ***Disease of nervous system*** |  |  |  |
| Encephalitis, myelitis, and encephalomyelitis | - | - | G04 |
| Anti-NMDA receptor encephalitis | - | - | G13.1 |
| Multiple sclerosis | 340 | 340 | G35 |
| Neuromyelitis optica and ADEM | 341 | 341A | G36 |
| Guillain-Barré syndrome | 354.01 | 357A | G61.0, G61.1, G61.8, G61.9 |
| Myasthenia gravis | 733.0 | 358A | G70.0 |
| ***Disease of the digestive system*** |  |  |  |
| Primary biliary cirrhosis | - | 571G | K74.3 |
| Crohn’s disease | 563.00 | 555 | K50 |
| Ulcerative colitis | 563.10, 569.02 | 556 | K51 |
| Coeliac disease | 269.00, 269.98 | 579A | K90.0 |
| **Others** |  |  |  |
| Acute rheumatic fever and chorea | 390-392 | 390-392 | I00, I01.0, I01.1, I01.2, I01.8, I01.9, I02.0, I02.9 |
| Sarcoidosis | 135 | 135 | D86 |
| IgA nephropaty | 580,582 | 580,582 | N00, N01,N03,N05 |
| ICD = International Classification of Diseases |  |  |  |

Supplementary Table 2. Background, pregnancy, and postpartum characteristics of the study population and the sibling cohort.

|  | **Study population** | |  |  | **Sibling** |  |
| --- | --- | --- | --- | --- | --- | --- |
| **Background characteristics** | **Women with PND** | | **Matched women without PND*** | | **Women with PND** | **Sister without PND** |
| n | 55299 |  | 552990 |  | 12469 | 14549 |
| Age at matching, mean (SD) | 30.7 | (5.58) | 30.7 | (5.58) | 30.51 (5.16) | 29.58 (5.12) |
| Period of delivery (%) |  |  |  |  |  |  |
| 2001-01-01 - 2006-06-01 | 13,747 | (24.9) | 138,462 | (25.0) | 3,077 (24.7) | 6,024 (41.4) |
| 2006-06-01 - 2009-02-09 | 13,991 | (25.3) | 138,071 | (25.0) | 3,265 (26.2) | 2,910 (20.0) |
| 2009-02-09 - 2011-07-19 | 13,785 | (24.9) | 138,324 | (25.0) | 3,145 (25.2) | 2,883 (19.8) |
| 2011-07-19 - 2013-12-31 | 13,776 | (24.9) | 138,133 | (25.0) | 2,982 (23.9) | 2,732 (18.8) |
| Maternal country of birth (%) |  |  |  |  |  |  |
| Scandinavian | 47,641 | (86.2) | 436,556 | (78.9) | 11,792 (94.6) | 13,589 (93.4) |
| Other | 7,658 | (13.8) | 116,434 | (21.0) | 677 (5.4) | 960 (6.6) |
| Maternal income at matching date (%) | |  |  |  |  |  |
| Low (<1064) | 10,304 | (18.6) | 111,019 | (20.1) | 2,257 (18.1) | 3,144 (21.6) |
| Middle (1064-2360) | 35,895 | (64.9) | 328,483 | (59.4) | 76,43 (61.3) | 8,560 (58.8) |
| High (>=2360) | 9,080 | (16.4) | 112,387 | (20.3) | 2,568 (20.6) | 2,835 (19.5) |
| Unknown | 20 | (0.0) | 1,101 | (0.2) | 1 (0.0) | 10 (0.1) |
| Maternal highest education at matching (%) | | |  |  |  |  |
| <=9 | 9,267 | (16.8) | 60,648 | (11.0) | 1,880 (15.1) | 1,899 (13.1) |
| 10 to 12 | 23,647 | (42.8) | 214,901 | (38.9) | 5,422 (43.5) | 6,481 (44.5) |
| >12 | 21,673 | (39.2) | 262,824 | (47.5) | 5,112 (41.0) | 6,085 (41.8) |
| Unknown | 712 | (1.3) | 14,617 | (2.6) | 55 (0.4) | 84 (0.6) |
| Early pregnancy BMI (%) |  |  |  |  |  |  |
| <18.5 | 1,283 | (2.3) | 12,267 | (2.2) | 304 (2.4) | 343 (2.4) |
| 18.5-25 | 27,296 | (49.4) | 303,521 | (54.9) | 6,367 (51.1) | 7,963 (54.7) |
| 25-30 | 12,920 | (23.4) | 123,388 | (22.3) | 2,828 (22.7) | 3,193 (21.9) |
| >30 | 8,214 | (14.9) | 59,465 | (10.8) | 1,785 (14.3) | 1,555 (10.7) |
| Unknown | 5,586 | (10.1) | 54,349 | (9.8) | 1,185 (9.5) | 1,495 (10.3) |
| Smoking three months prior to pregnancy (%) | | |  |  |  |  |
| Nonsmoker | 36,898 | (66.7) | 440,411 | (79.6) | 8,481 (68.0) | 10,563 (72.6) |
| 1-9 cigarettes/day | 6,364 | (11.5) | 43,388 | (7.8) | 1,392 (11.2) | 1,554 (10.7) |
| 10 cigarettes or more/day | 9,758 | (17.6) | 44,047 | (8.0) | 2,071 (16.6) | 1,788 (12.3) |
| Unknown | 2,279 | (4.1) | 25,144 | (4.5) | 525 (4.2) | 644 (4.4) |
| Family situation (%) |  |  |  |  |  |  |
| Married or cohabiting | 46,393 | (83.9) | 494,436 | (89.4) | 10,602 (85.0) | 12,869 (88.5) |
| Not married or cohabiting | 6,555 | (11.9) | 33,371 | (6.0) | 1,330 (10.7) | 1,013 (7.0) |
| Unknown | 2,351 | (4.3) | 25,183 | (4.6) | 537 (4.3) | 667 (4.6) |
| Region of residence in Sweden (%) | |  |  |  |  |  |
| South | 12,407 | (22.4) | 126,101 | (22.8) | 2,830 (22.7) | 3,333 (22.9) |
| Middle | 33,554 | (60.7) | 339,033 | (61.3) | 7,444 (59.7) | 8,641 (59.4) |
| North | 9,315 | (16.8) | 87,658 | (15.9) | 2,188 (17.5) | 2,569 (17.7) |
| Unknown | 23 | (0.0) | 198 | (0.0) | 7 (0.1) | 6 (0.0) |
| Parity (%) |  |  |  |  |  |  |
| First child | 28,443 | (51.4) | 250,932 | (45.4) | 6,385 (51.2) | 7,417 (51.0) |
| 2-3 children | 23,332 | (42.2) | 271,741 | (49.1) | 5,383 (43.2) | 6,479 (44.5) |
| ≥4 children | 3,524 | (6.4) | 30,317 | (5.5) | 701 (5.6) | 653 (4.5) |
| Psychiatric comorbidity (%) |  |  |  |  |  |  |
| No | 27,743 | (50.2) | 505,318 | (91.4) | 6,486 (52.0) | 12,788 (87.9) |
| Depression | 9,262 | (16.7) | 7,222 | (1.3) | 2,007 (16.1) | 273 (1.9) |
| Other psychiatric comorbidity | 18,294 | (33.1) | 40,450 | (7.3) | 3,976 (31.9) | 1,488 (10.2) |
| **Pregnancy** |  |  |  |  |  |  |
| Preeclampsia (%) | 2,019 | (3.7) | 15,166 | (2.7) | 427 (3.4) | 433 (3.0) |
| Gestational diabetes (%) | 808 | (1.5) | 5,929 | (1.1) | 146 (1.2) | 125 (0.9) |
| **Postpartum** |  |  |  |  |  |  |
| Length of gestation (%) |  |  |  |  |  |  |
| <32 weeks | 673 | (1.2) | 4,217 | (0.8) | 146 (1.2) | 107 (0.7) |
| 32-37 weeks | 3,263 | (5.9) | 22,523 | (4.1) | 704 (5.6) | 636 (4.4) |
| 37-41 weeks | 39,338 | (71.1) | 385,153 | (69.6) | 8,898 (71.4) | 9,981 (68.6) |
| >41 weeks | 10,090 | (18.2) | 119,506 | (21.6) | 2,300 (18.4) | 3,219 (22.1) |
| Unknown | 1,935 | (3.5) | 21,591 | (3.9) | 421 (3.4) | 606 (4.2) |
| Mode of delivery (%) |  |  |  |  |  |  |
| Vaginal | 38,367 | (69.4) | 419,877 | (75.9) | 8,771 (70.3) | 11,159 (76.7) |
| Instrumental vaginal | 4,449 | (8.0) | 41,946 | (7.6) | 998 (8.0) | 1,095 (7.5) |
| Cesarean section | 12,483 | (22.6) | 91,167 | (16.5) | 2,700 (21.7) | 2,295 (15.8) |
| Perinatal mortality (%) |  |  |  |  |  |  |
| Livebirth | 54,751 | (99.0) | 550,431 | (99.5) | 12,356 (99.1) | 14,506 (99.7) |
| Stillbirth | 398 | (0.7) | 1,796 | (0.3) | 80 (0.6) | 29 (0.2) |
| Infant death within 0-6 days | 106 | (0.2) | 563 | (0.1) | 23 (0.2) | 11 (0.1) |
| Infant death within 7-27 days | 44 | (0.1) | 200 | (0.0) | 10 (0.1) | 3 (0.0) |

PND = Perinatal depression, SD = standard deviation

*Women matched to PND women at the diagnosis date of PND.

Supplementary Table 3. Associations between (a) autoimmune disease and subsequent risk of perinatal depression and (b) perinatal depression and subsequent risk of autoimmune disease in all four models.

| 1. ***Autoimmune disease and subsequent risk of perinatal depression*** | | | | | | | | | | | | |
| --- | --- | --- | --- | --- | --- | --- | --- | --- | --- | --- | --- | --- |
| ***Matching set*** | **Model 0** | | | **Model 1** | | | **Model 2** | | | **Model 3** | | |
|  | OR | 95% CI | | aOR | 95% CI | | aOR | 95% CI | | aOR | 95% CI | |
| ***Population*** |  |  |  |  |  |  |  |  |  |  |  |  |
| PND | **1.55** | **(1.50,** | **1.60)** | **1.49** | **(1.44,** | **1.54)** | **1.30** | **(1.25,** | **1.35)** | **1.26** | **(1.21,** | **1.30)** |
| APD | **1.54** | **(1.47,** | **1.62)** | **1.47** | **(1.40,** | **1.54)** | **1.26** | **(1.19,** | **1.33)** | **1.21** | **(1.15,** | **1.28)** |
| PPD | **1.56** | **(1.49,** | **1.63)** | **1.52** | **(1.46,** | **1.60)** | **1.35** | **(1.28,** | **1.42)** | **1.31** | **(1.25,** | **1.38)** |
| ***Sibling*** |  |  |  |  |  |  |  |  |  |  |  |  |
| PND | **1.37** | **(1.24,** | **1.52)** | **1.37** | **(1.24,** | **1.52)** | **1.29** | **(1.15,** | **1.45)** | **1.26** | **(1.12,** | **1.42)** |
| APD | **1.30** | **(1.13,** | **1.50)** | **1.30** | **(1.12,** | **1.50)** | 1.18 | (1.00, | 1.41) | **1.19** | **(1.00,** | **1.41)** |
| PPD | **1.45** | **(1.25,** | **1.67)** | **1.46** | **(1.26,** | **1.68)** | **1.40** | **(1.18,** | **1.65)** | **1.37** | **(1.16,** | **1.62)** |
| 1. ***Perinatal depression and subsequent risk of autoimmune disease*** | | | | | | | | | | | | |
| ***Matching set*** | **Model 0** | | | **Model 1** | | | **Model 2** | | | **Model 3** | | |
|  | HR | 95% CI | | aHR | 95% CI | | aHR | 95% CI | | aHR | 95% CI | |
| ***Population*** |  |  |  |  |  |  |  |  |  |  |  |  |
| PND | **1.56** | **(1.50,** | **1.62)** | **1.52** | **(1.46,** | **1.58)** | **1.30** | **(1.25,** | **1.36)** | **1.28** | **(1.22,** | **1.34)** |
| APD | **1.56** | **(1.48,** | **1.63)** | **1.50** | **(1.43,** | **1.58)** | **1.27** | **(1.20,** | **1.35)** | **1.25** | **(1.18,** | **1.32)** |
| PPD | **1.57** | **(1.47,** | **1.68)** | **1.55** | **(1.45,** | **1.65)** | **1.35** | **(1.25,** | **1.46)** | **1.34** | **(1.24,** | **1.44)** |
| ***Sibling*** |  |  |  |  |  |  |  |  |  |  |  |  |
| PND | **1.45** | **(1.25,** | **1.67)** | **1.45** | **(1.25,** | **1.68)** | **1.33** | **(1.12,** | **1.57)** | **1.29** | **(1.08,** | **1.54)** |
| APD | **1.47** | **(1.23,** | **1.75)** | **1.49** | **(1.23,** | **1.80)** | **1.33** | **(1.07,** | **1.66)** | 1.25 | (0.99, | 1.57) |
| PPD | **1.42** | **(1.11,** | **1.82)** | **1.46** | **(1.13,** | **1.89)** | **1.44** | **(1.07,** | **1.95)** | **1.50** | **(1.10,** | **2.05)** |
| PND = Perinatal depression, APD = antepartum depression, PPD = postpartum depression, OR = Odds ratio, HR = Hazard ratio, CI = confidence interval  Model 0: Unadjusted model (the matching variables: age at delivery and year of delivery) | | | | | | | | | | | | |
| Model 1: the estimates were adjusted for maternal country of birth, region of residence, educational level, income, family situation, and parity | | | | | | | | | | | | |
| Model 2: the estimated were additionally adjusted for BMI, smoking, and psychiatric comorbidity | | | | | | | | | | | | |
| Model 3: the estimated were additionally adjusted for gestational diabetes, preeclampsia, gestational length, and mode of delivery | | | | | | | | | | | | |

Supplementary Table 4. Associations between (a) autoimmune disease and risk of subsequent perinatal depression and (b) perinatal depression and subsequent risk of autoimmune disease, stratified by calendar year at delivery, and maternal country of birth and parity.

| ***(a)   Autoimmune disease and subsequent risk of perinatal depression*** | | | | | | | | |
| --- | --- | --- | --- | --- | --- | --- | --- | --- |
|  | **PND** | | ***No PND**** | |  | | | *p* for interaction |
|  | n | AD, n (%) | n | AD, n (%) | aOR | 95% CI | |  |
| By calendar year at delivery |  |  |  |  |  |  |  |  |
| 2001-2009 | 32,564 | 2,217 (6.8) | 325,182 | 14,834 (4.6) | **1.29** | **(1.22,** | **1.36)** | 0.599 |
| 2010-2013 | 22,735 | 2,354 (10.4) | 227,808 | 15,605 (6.9) | **1.31** | **(1.25,** | **1.38)** |  |
| By maternal country of birth |  |  |  |  |  |  |  |  |
| Scandinavia | 47,641 | 4,040 (8.5) | 436,556 | 26,360 (6.0) | **1.27** | **(1.22,** | **1.32)** | **<0.001** |
| Other countries | 7,655 | 531 (6.9) | 116,379 | 4,079 (3.5) | **1.56** | **(1.40,** | **1.73)** |  |
| By parity |  |  |  |  |  |  |  |  |
| Primiparous | 28,443 | 2,258 (7.9) | 250,932 | 12,994 (5.2) | **1.29** | **(1.22,** | **1.36)** | 0.602 |
| Multiparous | 26,856 | 2313 (8.6) | 302,058 | 17445 (5.8) | **1.31** | **(1.25,** | **1.38)** |  |
| ***(b)   Perinatal depression and subsequent risk of autoimmune disease*** | | | | | | | | |
|  | ***Exposed to PND*** | | ***Unexposed to PND**** | |  |  |  | *p*-for interaction |
|  | n | AD, n (%) | n | AD, n (%) | aHR | 95% CI | |  |
| By calendar year at delivery |  |  |  |  |  |  |  |  |
| 2001-2009 | 30,347 | 2,221 (7.3) | 2893,84 | 13,562 (4.7) | **1.31** | **(1.25,** | **1.37)** | 0.671 |
| 2010-2013 | 20,381 | 807 (4.0) | 190,285 | 4,761 (2.5) | **1.28** | **(1.18,** | **1.39)** |  |
| By maternal country of birth |  |  |  |  |  |  |  | 0.799 |
| Scandinavia | 43,601 | 2,604 (6.0) | 376,514 | 14,545 (3.9) | **1.30** | **(1.24,** | **1.36)** |  |
| Other countries | 7,127 | 424 (5.9) | 103,155 | 3,778 (3.7) | **1.32** | **(1.18,** | **1.47)** |  |
| By parity |  |  |  |  |  |  |  |  |
| Primiparous | 26,185 | 1,625 (6.2) | 219,386 | 9,115 (4.2) | **1.24** | **(1.17,** | **1.32)** | **0.012** |
| Multiparous | 24,543 | 1,403 (5.7) | 260,283 | 9,208 (3.5) | **1.38** | **(1.29,** | **1.47)** |  |
| AD = autoimmune disease, PND = Perinatal depression, OR = Odds ratio, HR = Hazard ratio, CI = confidence interval  *Women matched to PND women at the diagnosis date of PND. | | | | | | | | |
| aOR: the estimates were adjusted for age and calendar year at matching, maternal country of birth, income, educational level, BMI, region of residence, smoking, family situation, parity, and psychiatric comorbidity | | | | | | | | |

Supplementary Table 5. Associations between (a) autoimmune disease and risk of subsequent perinatal depression and (b) perinatal depression and subsequent risk of autoimmune disease, by time of autoimmune disease to/from PND.

| ***(a)   Autoimmune disease and subsequent risk of perinatal depression*** | | | | | |
| --- | --- | --- | --- | --- | --- |
|  | **PND n (%)** | ***No PND****, n (%) | aOR | 95% CI | |
| no AD | 50,728 (91.7) | 522,569 (94.5) | ref | ref |  |
| 0-1 year from AD to match/PND | 784 (1.4) | 5,026 (0.9) | **1.43** | **(1.32,** | **1.56)** |
| 1-5 years from AD to match/PND | 1,485 (2.7) | 10,269 (1.9) | **1.31** | **(1.23,** | **1.39)** |
| 5-10 years from AD to match/PND | 1,127 (2.0) | 7,118 (1.3) | **1.33** | **(1.23,** | **1.43)** |
| >10 years from AD to match/PND | 1,175 (2.1) | 8,008 (1.4) | **1.19** | **(1.11,** | **1.28)** |
| ***(b)   Perinatal depression and subsequent risk of autoimmune disease*** | | | |  |  |
|  | ***Exposed to PND*** n (%) | ***Unexposed to PND****, n (%) | aHR | 95% CI | |
| no AD | 47,700 (94.0) | 422,119 (95.8) | ref | ref |  |
| 0-1 year from match/PND to AD | 937 (1.8) | 5,714 (1.3) | **1.59** | **(1.32,** | **1.93)** |
| 1-5 years from match/PND to AD | 1,403 (2.8) | 8,390 (1.9) | **1.24** | **(1.12,** | **1.38)** |
| 5-10 years from match/PND to AD | 620 (1.2) | 3,795 (0.9) | **1.31** | **(1.16,** | **1.47)** |
| >10 years from match/PND to AD | 68 (0.1) | 422 (0.1) | **1.53** | **(1.12,** | **2.08)** |
| AD = autoimmune disease. PND = Perinatal depression. OR = Odds ratio, HR = Hazard ratio, CI = confidence interval  * Women matched to PND women at the diagnosis date of PND. | | | | | |
| aOR: Adjusted for Maternal country of birth, income, educational level, BMI, region of residence, smoking, family situation, parity, and psychiatric comorbidity | | | | | |
| aHR: Adjusted for Maternal country of birth, income, educational level, BMI, region of residence, smoking, family situation, parity, and psychiatric comorbidity | | | | | |

Supplementary Table 6. Associations between (a) autoimmune disease and risk of subsequent perinatal depression and (b) perinatal depression and subsequent risk of autoimmune disease, considering the timing of the perinatal depression diagnosis

| ***(a)   Autoimmune disease and subsequent risk of perinatal depression^a.^*** | | | | | | | |
| --- | --- | --- | --- | --- | --- | --- | --- |
|  | ***PND*** | | ***No PND**** | |  |  | |
| **Matching set** | n | AD, n (%) | n | AD, n (%) | aOR | 95% CI | |
| APD 1st trimester | 15,606 | 1,267 (8.1) | 156,06 | 8,105 (5.2) | **1.29** | **(1.20,** | **1.38)** |
| APD 2nd trimester | 3,922 | 312 (8.0) | 39,22 | 2,111 (5.4) | **1.23** | **(1.06,** | **1.44)** |
| APD 3rd trimester | 1,9 | 155 (8.2) | 19 | 991 (5.2) | **1.55** | **(1.23,** | **1.95)** |
| PPD 0-3 months postpartum | 5,971 | 553 (9.3) | 59,71 | 3,807 (6.4) | **1.22** | **(1.10,** | **1.37)** |
| PPD 3-6 months postpartum | 6,216 | 586 (9.4) | 62,16 | 3,733 (6.0) | **1.42** | **(1.28,** | **1.57)** |
| PPD 6-12 months postpartum | 12,678 | 1,215 (9.6) | 126,78 | 8,133 (6.4) | **1.37** | **(1.28,** | **1.46)** |
| ***(b)   Perinatal depression and subsequent risk of autoimmune disease^b.^*** | | | | | | | |
|  | ***Exposed to PND*** | | ***Unexposed to PND**** | |  |  | |
| **Matching set** | n | AD, n (%) | n | AD, n (%) | aHR | 95% CI | |
| APD 1st trimester | 14,339 | 900 (6.3) | 135,983 | 5,476 (4.0) | **1.19** | **(1.10,** | **1.30)** |
| APD 2nd trimester | 3,61 | 240 (6.6) | 34,168 | 1,338 (3.9) | **1.51** | **(1.26,** | **1.80)** |
| APD 3rd trimester | 1,745 | 127 (7.3) | 16,549 | 685 (4.1) | **1.47** | **(1.13,** | **1.91)** |
| PPD 0-3 months postpartum | 5,418 | 220 (4.1) | 50,76 | 1,516 (3.0) | 1.09 | (0.92, | 1.29) |
| PPD 3-6 months postpartum | 5,63 | 281 (5.0) | 52,956 | 1,598 (3.0) | **1.47** | **(1.27,** | **1.71)** |
| PPD 6-12 months postpartum | 11,463 | 551 (4.8) | 107,359 | 3,199 (3.0) | **1.42** | **(1.28,** | **1.57)** |
| AD = autoimmune disease, PND = Perinatal depression, APD = antepartum depression, PPD = postpartum depression, OR = Odds ratio, HR = Hazard ratio, CI = confidence interval  * Women matched to PND women at the diagnosis date of PND | | | | | | | |
| aOR: Adjusted for Maternal country of birth, income, educational level, BMI, region of residence, smoking, family situation, parity, and psychiatric comorbidity | | | | | | | |
| aHR: Adjusted for Maternal country of birth, income, educational level, BMI, region of residence, smoking, family situation, parity, and psychiatric comorbidity | | | | | | | |
| a. 99 066 women excluded due to imputed date of diagnosis | | | | | | | |
| b. 50 261 women excluded due to imputed date of diagnosis | | | | | | | |

Supplementary Table 7. Associations between (a) autoimmune disease and risk of subsequent perinatal depression and (b) perinatal depression and subsequent risk of autoimmune disease, after excluding women with grief/stressors, and whose perinatal depression was defined only based on use of antidepressants.

| ***(a)   Autoimmune disease and subsequent risk of perinatal depression*** | | | | | | | |
| --- | --- | --- | --- | --- | --- | --- | --- |
|  | ***PND*** | | ***No PND**** | |  |  | |
|  | n | AD, n (%) | n | AD, n (%) | aOR | 95% CI | |
| Excluding women with stillbirth, neonatal death or very preterm birth | 54,236 | 4,456 (8.2) | 547,051 | 29,967 (5.5) | **1.30** | **(1.25,** | **1.35)** |
| Excluding PNDs classified by antidepressant prescription only | 19,178 | 1,313 (6.8) | 191,78 | 9,001 (4.7) | **1.17** | **(1.09,** | **1.26)** |
| ***(b)   Perinatal depression and subsequent risk of autoimmune disease*** | | | | | | | |
|  | ***Exposed to PND*** | | ***Unexposed to PND**** | |  |  | |
|  | n | AD, n (%) | n | AD, n (%) | aHR | 95% CI | |
| Excluding stillborn/neonatal death/premature | 49,78 | 2,952 (5.9) | 474,676 | 18,012 (3.8) | **1.30** | **(1.25,** | **1.36)** |
| Excluding PNDs classified by antidepressant prescription only | 19,543 | 1,406 (7.2) | 186,329 | 8,638 (4.6) | **1.30** | **(1.21,** | **1.39)** |
| AD = autoimmune disease, PND = Perinatal depression, OR = Odds ratio, HR = Hazard ratio, CI = confidence interval  *Women matched to PND women at the diagnosis date of PND | | | | | | | |
| aOR: Adjusted for Maternal country of birth, income, educational level, BMI, region of residence, smoking, family situation, parity, and psychiatric comorbidity | | | | | | | |
| aHR: Adjusted for Maternal country of birth, income, educational level, BMI, region of residence, smoking, family situation, parity, and psychiatric comorbidity | | | | | | | |
